# Early Remaining Useful Life Prediction of Lithium-Ion Batteries Based on a Hybrid Machine Learning Method with Time Series Augmentation

**DOI:** 10.3390/s26041238

**Published:** 2026-02-13

**Authors:** Jingwei Zhang, Jian Huang, Taihua Zhang, Erbao He, Sipeng Wang, Liguo Yao

**Affiliations:** 1 School of Mechanical and Electrical Engineering, Guizhou Normal University, Guiyang 550025, China; jingwz@gznu.edu.cn (J.Z.); zhangth542@gznu.edu.cn (T.Z.); 460141359@gznu.edu.cn (E.H.); lgyao@gznu.edu.cn (L.Y.); 2 Weining Autonomous County Vocational School, Bijie 553100, China; wnzzxzdzyxx@163.com; 3 Guizhou Key Laboratory of NewGen Cyberspace Security, Guizhou Normal University, Guiyang 550025, China; 4 Technical Engineering Center of Manufacturing Service and Knowledge Engineering, Guizhou Normal University, Guiyang 550025, China

**Keywords:** lithium-ion battery, early prediction, remaining useful life, time series generative adversarial network, dung beetle optimizer

## Abstract

Early and accurate prediction of the remaining useful life (RUL), defined as the number of operational cycles a battery can continue to function before reaching its end-of-life threshold, is crucial for improving the reliability of new energy vehicles. To address noise contamination, capacity regeneration effects, and data scarcity in early-stage prognostics, this paper proposes a hybrid framework integrating signal decomposition, time series augmentation, and deep forecasting. The raw capacity sequence is decomposed using Complete Ensemble Empirical Mode Decomposition with Adaptive Noise (CEEMDAN) to separate multi-scale components. A Transformer-enhanced time series generative adversarial network (HyT-GAN) is then employed to augment decomposed components, improving robustness under small-sample conditions. A CNN-BiGRU predictor is trained for capacity forecasting, and key hyperparameters are tuned via the Dung Beetle Optimizer (DBO). Experiments on NASA and CALCE benchmark datasets demonstrate that the proposed method achieves accurate early-stage prediction using only 20% historical data, with $R^{2}$ ranging from 0.9643 to 0.9972 and RMSE/MAE below 0.0296/0.0198. These results indicate that the proposed framework can deliver reliable RUL estimates under data-limited and noisy measurement conditions.

## 1. Introduction

Against the backdrop of escalating environmental degradation and energy crises, new energy vehicles are emerging as a dominant mode of transportation. This shift underscores the critical role of lithium-ion batteries (LIBs) performance monitoring [1,2]. As the primary energy source in electric vehicles, LIBs have attracted significant research focus on accurately assessing their state of health (SOH) and predicting their remaining useful life (RUL)—two critical functions that modern battery management systems require to ensure optimal performance [3]. LIBs, serving as the primary power source for modern energy storage and electrical equipment, offer notable advantages including compact size, lightweight design, high energy density, broad operating temperature ranges, extended cycle life, and low self-discharge rates [4]. However, repeated charge–discharge cycles induce irreversible electrochemical reactions within LIBs, leading to electrode material degradation and capacity fading. Significant performance degradation emerges as a critical indicator of battery aging, with the threshold for end of life (EOL) determination being reached once the achievable maximum discharge capacity persistently falls within 70% to 80% of its original rated capacity. Continued usage beyond this threshold risks equipment failure [5]. For battery-based systems to remain safe and reliable, it is vital to establish early RUL forecasting methodologies [6].

This paper proposes a hybrid machine learning framework for early-stage remaining useful life prediction of lithium-ion batteries. The proposed method is designed to address two key challenges in practice: (i) the performance degradation of single-feature models under small-sample conditions, and (ii) the difficulty of capturing capacity regeneration phenomena commonly observed in real-world battery degradation data.

First, the raw capacity sequence is decomposed using CEEMDAN to obtain multiple intrinsic mode functions (IMFs) and a residual component. The IMFs are expected to represent local fluctuations and regeneration-related behaviors, whereas the residual term characterizes the global degradation trend. Then, a HyT-GAN is employed to augment the decomposed components, thereby improving data diversity and enhancing the robustness of early-stage prediction.

After data augmentation, the reconstructed fusion data are fed into a CNN-BiGRU predictor to estimate the future capacity evolution. To adapt the predictive model to the heterogeneous characteristics of high-frequency and low-frequency components, the DBO is adopted to search the hyperparameter space and automatically configure the predictor. Finally, the overall RUL is obtained by aggregating the predicted results of all decomposed components. Experiments on two public benchmark datasets demonstrate that the proposed framework achieves superior performance compared with representative regression-based methods.

The main contributions of this paper are as shown below.

- A hybrid CEEMDAN–CNN-BiGRU model architecture based on DBO optimization is proposed. This framework addresses mode aliasing, improves data fidelity, adapts to heterogeneous degradation patterns through dynamic parameter optimization, and captures nonlinear battery aging dynamics.
- The time series generative adversarial network based on a Transformer module is introduced into the hybrid model to augment the data of decomposed IMF components, improve the robustness of the model in small-sample scenarios, and achieve the goal of early-stage RUL prediction.
- The experimental validation of this methodology employed cycling aging test data on lithium-ion cells curated by NASA’s Prognostics Center of Excellence and accelerated degradation datasets developed through the University of Maryland’s CALCE Battery Research Program, substantiating its capability to achieve superior remaining useful life estimation accuracy while maintaining robustness in data-constrained scenarios, with comparative analyses conducted against the prevailing regression-based prognostic approaches.

The structure of this paper is outlined below: Section 2 provides a literature review of the existing model-based and data-driven RUL prediction approaches and highlights the research gap motivating this study. Section 3 describes the proposed hybrid framework for early-stage RUL estimation, including the overall architecture and key modules. Section 4 details the experimental design, including the lithium-ion battery datasets, model settings, and evaluation metrics. Section 5 presents and discusses the early-stage RUL prediction results, including comparative experiments and ablation studies. Section 6 concludes the paper and outlines directions for future work.

## 2. Literature Review

Within the current academic literature, RUL prediction methodologies have been systematically classified by researchers into two principal frameworks: the model-based approach and the data-driven approach [7]. The model-based approach examines physicochemical phenomena and degradation mechanisms within battery systems to establish interpretable mathematical models capable of characterizing aging trends [8]. This methodology commonly integrates Kalman filter and particle filter algorithms to enhance the prognostic precision of remaining useful life predictions. To address prediction challenges, Chen et al. [9] formulated a combined linear optimization sampling particle filter methodology that incorporates the sliding window gray model within its computational framework. Vichard et al. [10] proposed a method combining a third-order equivalent circuit model with a Kalman filter for RUL prediction. Although model-based approaches have certain advantages in theory and do not require extensive degradation data [11], in practical applications, they are often limited by the model’s accuracy and the parameters’ availability. The complex and dynamic electrochemical mechanisms of battery systems pose challenges in precisely modeling degradation behavior throughout repeated cycling processes. Conversely, the data-driven approach has gained increasing attention in recent studies due to its effectiveness in processing big data and excellent generalization capacity [12].

The data-driven approach fundamentally employs artificial intelligence and statistical theory to uncover latent degradation signatures and predict the RUL through the analysis of historical battery cycling datasets. By circumventing dependence on lithium-ion electrochemical fundamentals, such approaches enable superior adaptability to field conditions while maintaining prediction robustness [13]. Machine learning-based approaches, owing to their strong nonlinear fitting capabilities, have become dominant in RUL prediction research. In addition to RUL prediction, machine learning methods can also support lithium-ion battery safety assessment [14]. Hu et al. [15] employed wavelet threshold denoising combined with a Transformer neural network for prediction, achieving effective RUL forecasting of LIBs. Cheng et al. [16] combined the backpropagation Long Short-Term Memory network (B-LSTM) with EMD, estimated the health state through the B-LSTM of the many-to-one structure, then used the neural network of the one-to-one structure to predict the RUL. Wang et al. [17] used Variational Mode Decomposition (VMD) for data processing and introduced the temporal convolutional neural network (TCN) with a self-attention mechanism to predict the RUL. However, the models proposed in the aforementioned studies require substantial historical data for training, and their prediction accuracy deteriorates as the training samples decrease, indicating potential reliability issues in practical applications.

Early-stage prediction of remaining useful life is critical for preventing unexpected failures in LIB systems. However, predicting the SOH and RUL estimation during early stages remains challenging. To address early RUL estimation in LIBs, Cai et al. [18] proposed a hybrid model for RUL prediction. The model decomposes the input capacity series with CEEMDAN and uses a Transformer network and deep neural network to predict the trend of components and residuals. The prediction results can be obtained using only 25–30% of historical data. Ma et al. [19] proposed a two-step method combining convolutional neural network and Gaussian process regression (GPR) to estimate the RUL. Tong et al. [20] introduced an innovative approach that integrates adaptive dropout LSTM with Monte Carlo simulation, enabling precise predictions with merely 25% of the historical data. Despite these advancements, the existing methods relying solely on historical capacity data as the input for early RUL prediction exhibit significant limitations. They demonstrate notable noise sensitivity, resulting in poor robustness of single-feature models that fail to capture capacity regeneration phenomena during battery degradation, ultimately leading to suboptimal SOH estimation accuracy. Furthermore, current approaches frequently encounter constrained model performance issues, preventing accurate RUL prediction when either reducing training data or increasing the total dataset size.

Multi-feature-based RUL prediction approaches, utilizing diverse health indicators (HIs), have become prevalent in early prediction. In LIB systems, RUL can be predicted by establishing the regression relationships between informative measurements (e.g., charge/discharge voltage, charge/discharge current, and impedance) and capacity degradation [21,22]. Liang et al. [23] proposed an early prediction method based on the state space model, using the IQR method to identify and correct abnormal data. Lv et al. [24] used CEEMDAN to decompose the HIs. Then, the decomposed components are input into CNN-BiGRU model for prediction. While current multi-feature RUL prediction frameworks utilizing HIs demonstrate enhanced accuracy in early applications, most HIs exhibit limited physicochemical interpretability. Practical implementation requires synchronized multi-sensor data acquisition, which becomes challenging in real-world scenarios. Sensor drift-induced anomalous noise degrades prediction precision. Additionally, high-dimensional inputs escalate computational complexity, imposing stringent demands on prediction models. Such inherent limitations may ultimately undermine the reliability of early RUL prediction systems.

However, robust early-stage RUL prediction remains insufficiently addressed. Capacity-only methods are noise-sensitive and may miss capacity regeneration, while multi-feature approaches increase deployment difficulty due to multi-sensor requirements, drift, and computational burden. Moreover, deep models can be unstable with scarce data and sensitive to hyperparameter choices. Therefore, we propose a unified framework combining CEEMDAN decomposition, HyT-GAN augmentation, CNN-BiGRU prediction, and DBO-based hyperparameter optimization to improve robustness and early-stage generalization.

## 3. Methodology

### 3.1. CEEMDAN Decomposition

During the battery data acquisition process, environmental noise interference and capacity regeneration effects often introduce significant noise signals into the raw dataset, which can substantially degrade model prediction accuracy. To address this issue, researchers have developed a series of signal processing methods. The employed EMD algorithm [25] can extract degradation trend features from battery capacity sequences, but its inherent limitation lies in the tendency to produce mode mixing during the decomposition process. To overcome this drawback, the subsequently proposed Ensemble Empirical Mode Decomposition (EEMD) method [26] suppresses mode mixing by repeatedly introducing white noise into the signal. However, this approach results in residual Gaussian noise during signal reconstruction.

The CEEMDAN technique builds upon the advantages of both EMD and EEMD while introducing critical improvements: first, it retains the core concept of adding Gaussian noise from EEMD; second, it adopts a stepwise iterative strategy—after solving each IMF component, white noise is reintroduced into the residual signal, followed by multiple rounds of averaging. This enhanced approach not only improves the computational efficiency but also significantly enhances the signal reconstruction quality. The IMF components obtained through this method fully preserve the characteristics of the original signal. The decomposed IMFs undergo data augmentation via HyT-GAN before serving as inputs to the CNN-BiGRU network. The specific decomposition process of CEEMDAN is as follows:

Step 1: Parameter Initialization and Generate Noisy Signal Ensemble.

Define the original signal to be decomposed as x(t), where t∈[1,T]. Add adaptive noise:

(1) $$x^{\left(k\right)}(t)=x(t)+\varepsilon \cdot w^{\left(k\right)}(t)$$

Step 2: Compute the First IMF Component (*IMF*_1_):

(2) $${IMF}_{1}\left(t\right)=\frac{1}{N}\sum_{i=1}^{N}{IMF}_{1}^{k}(t)$$

(3) $$r_{1}\left(t\right)=x\left(t\right)-{IMF}_{1}(t)$$

Step 3: Iteratively Compute Higher-Order IMF Components (*IMFᵢ*, *i* ≥ 2).

For each order *i* ≥ 2, repeat the following steps until termination criteria are met:

Construct Noisy Residual Signal:

(4) $$r_{i-1}^{\left(k\right)}(t)=r_{i-1}(t)+\varepsilon_{i-1}\cdot E_{i-1}(w^{\left(k\right)}(t))$$

where $E_{i-1}(\cdot )$ denotes the residual after decomposing the noise into the (*i* − 1)th IMF, and $\varepsilon_{i-1}$ is the adaptively adjusted noise coefficient.

Decompose Noisy Residual: Perform EMD on $r_{i-1}^{\left(k\right)}(t)$ to extract its first-order component ${IMF}_{i}^{k}(t)$.

Ensemble Averaging and Update Residual:

(5) $${IMF}_{i}\left(t\right)=\frac{1}{N}\sum_{i=1}^{N}{IMF}_{i}^{k}(t)$$

(6) $$r_{i}\left(t\right)=x\left(t\right)-{IMF}_{i}(t)$$

The iteration stops when the residual $r_{i}\left(t\right)$ becomes monotonic or the preset maximum order *K* is reached.

Step 4: Output Decomposition Results.

The original signal can be reconstructed as

(7) $$x\left(t\right)=\sum_{i=1}^{K}{IMF}_{1}^{j}\left(t\right)+r_{K}\left(t\right)$$

where $r_{K}\left(t\right)$ represents the final residual, capturing the long-term trend or residual noise.

### 3.2. HyT-GAN Model for Data Augmentation

The proposed time series generative adversarial network with a hybrid Transformer module (HyT-GAN) performs high-fidelity time series data augmentation. The key improvements in HyT-GAN implementation over the original GAN lie primarily in its Transformer-enhanced hybrid architecture and domain-specific optimizations for time series forecasting. A Generative Adversarial Network represents a deep learning framework introduced by Ian Goodfellow et al. [27]. The framework comprises two competing neural architectures, a generator (*G*) and a discriminator (*D*), which participate in a minimax optimization process wherein *G* produces statistically credible synthetic data, while *D* conducts authenticity discrimination through binary classification of the generated instances. When applied to lithium-ion battery RUL prediction, GANs can approximate continuous degradation trajectories by learning the true data manifold. The generator generates reasonable data points from latent vectors z ∈ Z. The following equation can describe the function of the generator:

(8) $$G\left(z;\theta_{g}\right)$$

where *z* is the input noise and $\theta_{g}$ is the generator model parameter. The discriminator outputs a scalar representing the true probability of the input data. The discriminator’s working mechanism can be expressed using the mathematical expression below:

(9) $$D\left(x;\theta_{d}\right)$$

#### 3.2.1. GAN Model Training Process

The learning mechanism of GAN operates through an adversarial minimax framework established between generator network *G* and discriminator network *D*. Specifically, the generator’s objective focuses on producing artificial data distributions that precisely replicate the characteristics of genuine data distribution $p_{data}(x)$, whereas the discriminator’s function involves accurately differentiating original instances drawn from $p_{data}(x)$ from artificial outputs created through the generator’s synthesis process. This adversarial dynamic is formalized through a joint optimization objective:

The discriminator *D*, parameterized by $\theta_{D}$, is trained to maximize its ability to classify real and generated data. Its loss function combines two logarithmic expectations:

(10) $$L_{D}=E_{x\sim p_{data}(x)}\left[\log D(x)\right]+E_{z\sim p_{z}(z)}\left[\log(1-D(G(z)))\right]$$

where the first term $E_{x}\left[\log D(x)\right]$ quantifies the discriminator’s confidence in recognizing real data, while the second term $E_{z}\left[\log(1-D(G(z)))\right]$ measures its accuracy in rejecting synthetic samples. Maximizing $L_{D}$ sharpens the discriminator’s decision boundary between real and fake distributions.

Conversely, the generator *G*, parameterized by $\theta_{G},$ seeks to minimize the discriminator’s classification accuracy by producing data that *D* misclassifies as real. This is achieved by minimizing

(11) $$L_{G}=E_{z\sim p_{z}(z)}\left[\log-D(G(z))\right]$$

Equivalently, *G* aims to maximize $E_{z}\left[\log D(G(z))\right]$, driving the generated distribution $p_{G}$ toward alignment with $p_{data}(x)$.

The training alternates between updating $\theta_{D}$ (with *G* fixed) and $\theta_{G}$(with *D* fixed), forming a Nash equilibrium-seeking process. The equilibrium is achieved when $p_{G}=p_{data}$, at which point D(x)=0.5 for all samples, indicating indistinguishable real and synthetic distributions.

For temporal data generation in battery RUL prediction, the generator employs a Transformer-based architecture to model long-range dependencies in capacity degradation sequences. The self-attention mechanism enables global interaction across time steps, ensuring temporal consistency in synthesized trajectories. The discriminator combines convolutional layers for local pattern extraction and self-attention for sequence-level authenticity assessment, enforcing both local realism and global coherence in generated samples.

#### 3.2.2. GAN Model Based on Transformer Module

**Figure 1:**
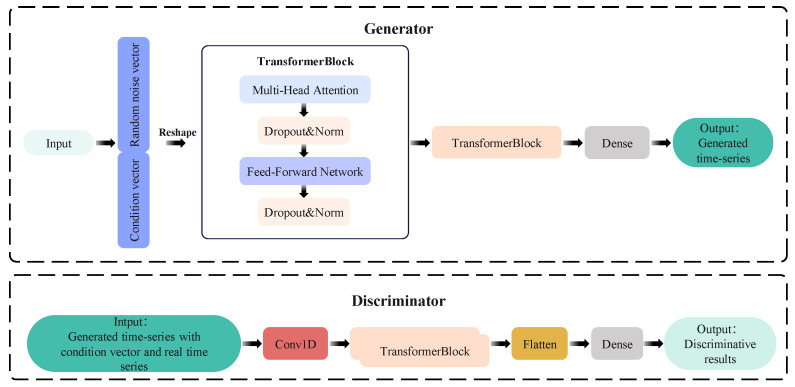
The structure of the HyT-GAN model.

Effective early RUL prediction relies on constructing high-quality time series, which necessitates the careful consideration of two critical factors: how past and future data points correlate within each cycle, and how different cycles exhibit both consistent and divergent patterns over time. The time series generative adversarial network designed with a Transformer module ensures these characteristics. Potential vectors are mapped into synthetic time series data through stacked Transformer blocks in the generator, while the discriminator combines local feature extraction and global dependency modeling to distinguish true and false samples. Its structure is shown in Figure 1.

The Transformer block is the basic unit of the model. The design uses the self-attention mechanism to model the sequence dependency globally and uses the residual structure to alleviate the gradient disappearance problem, forming a stackable feature enhancement module. Its structure includes the following core modules:

Multi-head self-attention layer: Through parallel computing of multiple independent attention heads, the association patterns of different subspaces of the input sequence are learned, respectively, and finally stitched and linearly projected into the comprehensive attention feature. The multi-head self-attention mechanism is formulated as

(12) $$Attention\left(Q,K,V\right)=softmax(\frac{QK^{T}}{\sqrt{d_{k}}})V$$

where *Q*, *K*, and *V* denote the query, key, and value matrices, respectively; and *d_k_* represent the dimension of keys.

Feed-forward network: It is composed of two full connection layers, and the Rectified Linear Unit (ReLU) activation function is used to enhance the nonlinear expression ability.

Residual connection and layer normalization: The original information is retained through element-level addition, and layer normalization is used to accelerate the convergence.

Dropout: Dropout layers are incorporated after both self-attention and feed-forward network outputs, with an empirically determined rate of 0.1 to mitigate overfitting.

The generator’s fundamental advancement centers on embedding the Transformer’s attention-weighting system within adversarial learning frameworks. Its processing flow is as follows: first, the input vector is mapped to the Transformer’s embedding dimension through the full connection layer, and the embedded vector is converted to the sequence format using the reshape operation to adapt to the Transformer’s sequence input requirements. By stacking two Transformer blocks, the global statistical characteristics of the sequence are learned layer by layer. Finally, the feature is mapped to the target sequence dimension through the full connection layer to generate a composite sequence that conforms to the real data distribution.

In the discriminator, the input sequence first extracts local pattern features through one-dimensional convolution. Two Transformer blocks with the same structure as the generator are stacked, and the sequence’s local features and global context information are fused through the self-attention mechanism. Flatten the Transformer output and send it to the full connection layer (Sigmoid activation). The probability that the sample output is real data.

### 3.3. CNN-BiGRU Prediction Model Based on DBO Optimization

#### 3.3.1. CNN-BiGRU Model

The IMF component after data augmentation is input into the CNN-BiGRU model for prediction. In the CNN module, the input data first traverses a 1-D convolutional layer with kernels sliding along the temporal dimension. The nonlinear representation capability is strengthened through the application of the ReLU activation function, as mathematically expressed in Equation (13). Subsequently, a 1-D max-pooling operation (Equation (14)) is performed on the generated features. This processing stage serves dual purposes: it facilitates the extraction of salient features from the convolutional layer’s output while simultaneously achieving parameter reduction. Consequently, the model gains enhanced computational efficiency with mitigated overfitting risks.

(13) $$y^{t}=Relu\;(b^{t}+\sum_{i=1}^{k}W_{i}^{t}*x^{t-1})$$

(14) $$q^{t}=Maxpool\;\left(y^{t}\right)$$

where *x^t−^*^1^ denotes the feature input set for the (*t* − 1) th convolutional module. Specifically, at the network’s initial stage (when *t* = 1), this variable corresponds to the primitive input capacity tensor. The term *b^t^* quantifies the bias adjustment component, while $W_{i}^{t}$ designates the parameter matrix of convolution kernels in the *t*-th layer. The hyperparameter *k* specifies the cardinality of filter kernels, symbol * represents the convolution operation, and *q^t^* is the result of pooling *y^t^*.

**Figure 2:**
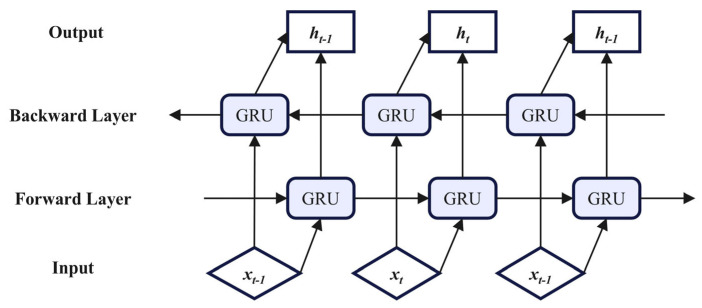
BiGRU network structure.

The exclusive reliance on CNN for feature extraction may fail to capture long-term temporal dependencies in sequential data. To address this limitation, a BiGRU is incorporated into the framework. GRU is an improved recurrent neural network (RNN) structure, which is used to improve the gradient vanishing/explosion problem of traditional RNNs when processing long sequence data. The GRU controls the flow of information by introducing two gates (update gate and reset gate), thereby improving the ability of the model to process sequence data. Compared with RNN, GRU is simpler in structure, more computationally efficient and equally excellent in processing time series data. The adoption of BiGRU further enhances the capability to capture temporal variations in data, where its bidirectional architecture simultaneously processes past and future contextual information. This design strengthens generalization across heterogeneous sequence lengths and structures, while achieving precise holistic temporal dependency modeling and accurate feature vector extraction. The specific structure of the BiGRU network is shown in Figure 2.

The output of the BiGRU neural network is shown in the formula:

(15) $$\left\{\begin{array}{c} \vec{h_{t}}=GRU(x_{t},\vec{h_{t-1}})\\ \overset{\leftarrow }{h_{t}}=GRU(x_{t},\overset{\leftarrow }{h_{t-1}})\\ h_{t}=\alpha_{t}\vec{h_{t}}+\beta_{t}\overset{\leftarrow }{h_{t}}+b_{t} \end{array}\right.$$

where *GRU* (*·*) denotes the gated recurrent unit; $\underset{h_{t}}{\to }$ and $\underset{h_{t}}{\leftarrow }$ correspond to the hidden state outputs generated by the forward-propagating layer and reverse-propagating layer, respectively. The parameters $\alpha_{t}$ and $\beta_{t}$ denote the attention weights assigned to the hidden states of the frontward-directional layer and backward-directional layer, while $b_{t}$ indicates the bias term added during computation.

In the hybrid model, a multilayer perceptron (MLP) is applied to extract and transform features in the CNN and BiGRU layers, further integrating and making decisions on these features and outputting the prediction results through the fully connected layer. These features are subsequently integrated through a series of fully connected (FC) layers: (1) the first FC layer introduces nonlinearity via ReLU activation, enabling complex feature combination learning; (2) the intermediate FC layer reduces feature dimensionality to enhance generalization; (3) the final FC layer serves as the regression head, mapping the processed features to continuous RUL predictions. This hierarchical structure accomplishes end-to-end feature-to-decision transformation.

#### 3.3.2. Dung Beetle Optimizer

The Dung Beetle Optimizer, a swarm intelligence algorithm originally introduced in 2022 [28], replicates five characteristic behaviors observed in dung beetles: rolling, dancing, foraging, breeding, and stealing. Its population is structured into four specialized categories: rolling beetles, breeding beetles, foraging beetles, and stealing beetles, with each category linked to defined optimization processes. Furthermore, the Dung Beetle Optimizer has been applied to hyperparameter optimization tasks in deep learning-based wind power forecasting models, where improved variants achieved substantial enhancements in prediction performance [29].

(1) Rolling beetles

This component emulates the trajectory-planning behavior of dung beetles through a celestial navigation framework. The positional update mechanism operates as

(16) $$x_{n}\left(t+1\right)=x_{n}\left(t\right)+\eta \times k\times x_{n}\left(t-1\right)+\delta \times \Delta x$$

(17) $$\Delta x=\left|x_{n}\left(t\right)-X^{w}\right|$$

In the formula, t represents the current number of iterations. $x_{n}\left(t\right)$ represents the position information of the n-th beetle at iteration t. η is a path deviation coefficient (probabilistically assigned −1 or 1). It is assigned to a value of −1 or 1 based on probabilistic methods. *k* ∈ (0, 0.2) represents the deflection coefficient, and δ ∈ (0, 1) denotes a constant. $X^{w}$ is the global worst position. Δx is used to simulate variations in light intensity.

When the dung beetle encounters obstacles and cannot move forward, it needs to reposition by dancing.

(18) $$x_{n}\left(t+1\right)=x_{n}\left(t\right)+\tan\left(\theta \right)\left|x_{n}\left(t\right)-x_{n}\left(t-1\right)\right|$$

In the formula, $\theta \in \left[0,\pi \right]$ represents the deflection angle. When $\theta =\left\{0,\;\frac{\pi }{2}\;or\;\pi \right\}$, the dung beetle’s position remains unchanged.

(2) Breeding beetles

A boundary-constrained strategy defines the oviposition region:

(19) $${Lb}^{*}=\max\left(X^{*}\times \left(1-R\right),Lb\right),{Ub}^{*}=\min\left(X^{*}\times \left(1-R\right),Ub\right)$$

In the formula, $X^{*}$ represents the current local optimum. ${Lb}^{*}$ and ${Ub}^{*}$ represent the dynamic oviposition boundaries. Where $R=1-\frac{t}{T_{max}}$, $T_{max}$ is the maximum iteration. Lb and Ub are the original problem bounds.

During the iterative process, the position of the brood ball is dynamically updated and defined as

(20) $$B_{n}\left(t+1\right)=X^{*}+b_{1}\times \left(B_{n}\left(t\right)-{Lb}^{*}\right)+b_{2}{\times (B}_{n}\left(t\right)-{Ub}^{*})$$

where $B_{n}\left(t\right)$ represents the *n*-th brood ball’s position at iteration *t*, with *b*_1_ and *b*_2_ as 1 × D independent random vectors (D: problem dimensionality).

(3) Foraging beetles

An optimal foraging zone is established to simulate the foraging behavior of small dung beetles, where the optimal foraging zone is defined as

(21) $${Lb}^{b}=\max\left(X^{b}\times \left(1-R\right),Lb\right),{Ub}^{b}=\min\left(X^{b}\times \left(1-R\right),Ub\right)$$

In the formula, $X^{b}$ represents the global optimum position. ${Lb}^{b}$ and ${Ub}^{b}$ represent foraging region boundaries. The position update for foraging dung beetles is defined as follows:

(22) $$x_{n}\left(t+1\right)=x_{n}\left(t\right)+C_{1}\times \left(x_{n}\left(t\right)-{Lb}^{b}\right)=C_{2}\times (x_{n}\left(t\right)-{Ub}^{b})$$

In the formula, $C_{1}$ is a normally distributed random variable and $C_{2}\in \left[0,1\right]$ denotes a random vector.

(4) Stealing beetles

Some dung beetles steal dung balls from others, and their stealing behavior pattern is updated as follows:

(23) $$x_{n}\left(t+1\right)=X^{b}+\mu \times \varepsilon \times \left(\left|x_{n}\left(t\right)-X^{*}\right|\right)+\left|x_{n}\left(t\right)-X^{b}\right|)$$

where ε is a normally distributed random vector, and μ serves as a constant scaling factor.

#### 3.3.3. Hyperparameter Optimization Process

In the CNN-BiGRU model, hyperparameters must be predefined as they determine the neural network architecture and cannot be learned from data. The hyperparameters in the neural network model are fed into the DBO algorithm, and the optimal hyperparameters are calculated by setting the objective function as the judgment basis through repeated iterative optimization. The detailed steps for optimizing the hyperparameters of the dung beetle algorithm are as follows:

(1) The population is initialized randomly to determine the population size, and the optimal path is obtained according to the number of iterations and the generated random number. After evaluating fitness function values, the best-performing hyperparameters are fed into the neural network model.
(2) The neural network is trained with the optimized hyperparameters, where each IMF decomposed by CEEMDAN is trained separately.
(3) Calculate the output loss function, update the weight through the gradient descent principle, and realize the multiple iterative calculations of the CNN-BiGRU model so that the prediction model gradually converges.
(4) After several iterations, the dataset is tested to judge the prediction performance of the model.
(5) According to the evaluation results, the population number, iteration times and other parameters in the DBO algorithm are adjusted accordingly.
(6) Repeat steps (1)~(5) until the neural network model with the best performance is obtained.

The hyperparameter optimization process of the dung beetle algorithm is shown in Algorithm 1.
**Algorithm 1.** Dung Beetle Optimizer (DBO) for hyperparameter optimizationInput: Population size (*N*), Max iterations (*T*), *LB*, UB, Neural network model, Training/validation data for each CEEMDAN component {IMF_k} Output: Best hyperparameters X_best1.Initialize population {X_i} (i = 1…N) uniformly within [LB,UB]2.Evaluate initial fitness of each X_i3.Set X_best as the best candidate in the initial population4.for t = 1 to T do5.        for each candidate X_i do6.                for each IMF_k do7.                        Build CNN-BiGRU8.                        Train on IMF_k training set using batch size b9.                        Compute validation RMSE_k(X_i)10.                end for11.                Fitness_i = mean_k RMSE_k(X_i)12.        end for13.        Update positions {X_i} using DBO update rules14.        Apply boundary handling and integer rounding for discrete variables15.end for16.Return X_best

### 3.4. Structure and Workflow of Hybrid Model

**Figure 3:**
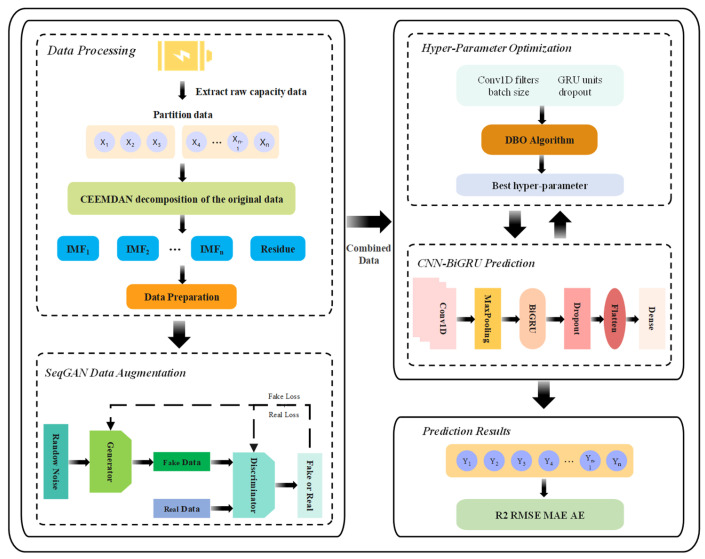
Framework of hybrid machine learning method with time series augmentation. The ellipses (“…”) indicate intermediate IMFs/samples omitted for brevity (i.e., IMF_1_, IMF_2_, …, IMF_n_ and x_1_, x_2_, …, x_n_).

The proposed lithium-ion battery RUL prediction framework adopts an end-to-end hybrid architecture that integrates signal decomposition, time series data augmentation, deep learning-based capacity forecasting, and hyperparameter optimization. As illustrated in Figure 3, raw capacity measurements acquired from sensors are first decomposed into multiple IMFs and a residual component using CEEMDAN. To alleviate data scarcity in early prediction scenarios, the decomposed components are augmented via a HyT-GAN. The augmented sequences are then fed into a hybrid CNN-BiGRU model for capacity prediction, whose key hyperparameters are automatically tuned by the Dung Beetle Optimizer to ensure optimal performance. Finally, the predicted capacity trajectory is reconstructed and used to estimate the remaining useful life based on a predefined degradation threshold.

Specifically, CEEMDAN is employed to handle the non-stationary and noisy nature of sensor-acquired capacity data by decomposing the original signal into multiple IMFs, where high-frequency components mainly capture short-term fluctuations and reversible capacity recovery, while low-frequency components and residuals represent long-term irreversible degradation trends. Each decomposed component is divided into training and testing subsets, and the training data are augmented using HyT-GAN, in which a Transformer-based generator learns global temporal dependencies and produces synthetic sequences consistent with real degradation patterns, while the discriminator combines convolutional feature extraction with self-attention to distinguish real and generated samples. The augmented data are subsequently used to train a hybrid CNN-BiGRU model, where convolutional layers extract local temporal features and the bidirectional gated recurrent unit captures long-range dependencies in both the forward and backward directions. During training, the DBO algorithm optimizes the critical hyperparameters, including learning rate, batch size, CNN filter numbers, and BiGRU hidden units. The predicted capacity of each component is finally reconstructed to obtain the overall capacity trajectory, from which the battery RUL is determined when the capacity reaches the predefined end-of-life threshold.

## 4. Experimental Setup

### 4.1. Source of Data

This paper utilizes two public-standard electrochemical datasets from NASA PCoE, CALCE and Oxford Battery Degradation Dataset as experimental substrates for prognostic model validation [30,31,32]. Due to their strong feasibility and applicability, these datasets are widely used to verify and evaluate the performance of battery prognostics algorithms. The data used in this paper are openly available. The NASA PCoE battery dataset can be accessed at https://www.nasa.gov/content/prognostics-center-of-excellence-data-set-repository (accessed on 8 May 2025), the CALCE CS2 dataset is available at https://calce.umd.edu/data#CS2 (accessed on 8 May 2025), and the Oxford Battery Dataset is available at https://ora.ox.ac.uk/objects/uuid:03ba4b01-cfed-46d3-9b1a-7d4a7bdf6fac (accessed on 6 January 2026).

The NASA dataset contains nine cyclically stressed 18,650 cell groups (2.0Ah nominal capacity) subjected to accelerated aging protocols. This paper compares the accuracy of the proposed RUL prediction model applied to batches 5, 6, and 7. The technical parameters of NASA’s chosen lithium-ion battery are presented in detail within Table 1.

The CALCE CS2 dataset comprises lithium-ion battery records featuring a nominal capacity of 1.1 Ah. In this paper, the batteries numbered 35, 36, and 37 were used to evaluate the performance of our early prediction method. The EOL criterion of the test is that the capacity decreases from 1.1 Ah to 0.77 Ah. The detailed specifications are shown in Table 2.

The Oxford Battery Degradation Dataset 1 contains long-term cycling measurements of eight commercial Kokam (SLPB533459H4) lithium-ion pouch cells with a nominal capacity of 740 mAh, all tested in a thermal chamber at 40 °C. In the aging protocol, each cell undergoes repeated drive cycle aging blocks in which the cells are charged under a CC–CV profile and discharged under a variable-current load derived from the Artemis Urban driving profile. To provide consistent reference measurements over the entire aging trajectory, characterization tests are performed periodically (every 100 drive cycles), consisting of 1C charge/discharge cycles (current = 740 mA) and pseudo-OCV tests (current = 40 mA). In this study, Cells 1, 3, and 7 from the Oxford dataset are selected to evaluate the effectiveness and generalization of the proposed early-stage RUL prediction framework across different degradation trajectories.

**Figure 4:**
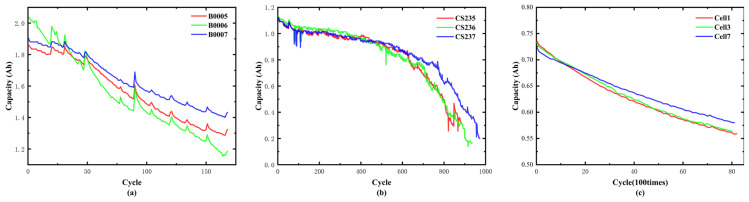
Capacity curve of dataset: (**a**) NASA; (**b**) CALCE; (**c**) Oxford.

The RUL curve of the battery between the NASA, CALCE and Oxford datasets is presented in Figure 4.

### 4.2. Model Setting

#### 4.2.1. Early-Stage Protocol and Input Configuration

The proposed framework uses historical capacity trajectories as the only input feature. Following common practice in the RUL literature, we evaluate both a standard setting and an early-stage setting. Specifically, 50% of the available historical capacity data is used as the prediction starting point for standard benchmarking under identical cycling conditions, while a reduced-input scenario using 20% of historical data is further adopted to assess early-stage prediction capability [33].

A sliding window strategy is employed to construct supervised samples. Given a look-back window length L, the model uses the past L capacity values to predict the capacity at the next cycle (one-step-ahead forecasting). Rolling prediction is then performed to obtain the future capacity trajectory until reaching the predefined end-of-life threshold.

#### 4.2.2. HyT-GAN Architecture and Key Hyperparameters

To alleviate data scarcity in early-stage scenarios, each CEEMDAN-decomposed component is augmented using HyT-GAN. In our implementation, the GAN is trained on sequence samples constructed by concatenating the sliding window input and the next-step target, i.e., $s=[x_{t-L+1:t},y_{t+1}]\in R^{(L+1)\times 1}$. The input capacity values are scaled to $\left[-1,1\right]$ prior to GAN training to match the tanh output range of the generator.

Generator G: The generator takes a Gaussian latent vector $z\in R^{64}$ and projects it to a sequence embedding via a dense layer, followed by reshaping into $(L+1)\times d_{\mathrm{model}}$. The sequence is then processed by N=2 stacked Transformer blocks (multi-head self-attention + feed-forward network + residual connections + layer normalization). A time-distributed dense layer outputs a synthetic sequence $\hat{s}\in R^{(L+1)\times 1}$ with tanh activation. Key hyperparameters: Latent dimension =64; $d_{\mathrm{model}}=64$; attention heads h=4; feed-forward dimension $d_{\mathrm{ff}}=128$; Transformer blocks N=2; Transformer dropout rate =0.1.

Discriminator D: The discriminator receives a real or generated sequence $s\in R^{(L+1)\times 1}$. It first applies a Conv1D feature extractor to capture local patterns and then uses the same Transformer configuration ($N=2,\;\;h=4,{\;d}_{\mathrm{model}}=64,\;\;d_{\mathrm{ff}}=128$) to model global dependencies. Global average pooling is used to aggregate temporal features, followed by a sigmoid output for real/fake discrimination. Key hyperparameters: Conv1D filters =64, kernel size =3 (padding “same”); Transformer blocks N=2 with h=4, $d_{\mathrm{model}}=64$, $d_{\mathrm{ff}}=128$.

#### 4.2.3. Loss Functions and Loss Convergence Criteria

HyT-GAN adversarial losses: HyT-GAN is trained using the standard binary cross-entropy (BCE) objective. Let D(⋅)∈(0,1) be the discriminator output. The discriminator is trained to classify real sequences as 1 and generated sequences as 0, while the generator is trained to fool the discriminator. The losses are formulated as

(24) $$L_{D}=BCE(y_{r},D(s))+BCE(0,D(G(z))),L_{G}=BCE(1,D(G(z)))$$

where label smoothing is applied to real labels with $y_{r}=0.9$. Both G and D are optimized using Adam with learning rate $2\times 10^{-4}$, $\beta_{1}=0.5$, and $\epsilon =10^{-7}$. HyT-GAN is trained for 200 epochs with batch size 128, and to ensure stable adversarial updates under small-sample settings, each epoch uses a fixed number of 20 training steps (randomly sampling real sequences per step).

Training stability/convergence criterion: In our implementation, HyT-GAN runs for a fixed number of epochs, and convergence is assessed by monitoring $L_{D}$ and $L_{G}$ across epochs. Training is considered stable when both losses remain finite and show a clear plateau trend in the later epochs (i.e., no divergence). To further improve numerical stability, global-norm gradient clipping is applied with clip value =5.0. After training, the generator is used to synthesize additional $\left(L+1\right)$-length sequences, which are then split into $\left(x,y\right)$ pairs for downstream predictor training.

CNN-BiGRU prediction loss: The capacity predictor is trained using mean squared error:

(25) $$L_{\mathrm{pred}}=\frac{1}{n}\sum_{i=1}^{n}({\hat{y}}_{i}-y_{i}{)}^{2}$$

where $y_{i}$ and ${\hat{y}}_{i}$ denote the ground-truth and predicted capacity at cycle i.

#### 4.2.4. CNN-BiGRU Predictor Architecture and Hyperparameter Optimization

After augmentation, the forecasting network adopts a hybrid CNN-BiGRU architecture. A Conv1D layer extracts local temporal patterns from the input window, while a bidirectional GRU captures long-range dependencies in both the forward and backward directions. Dropout is applied to mitigate overfitting, and a final dense layer outputs the one-step-ahead capacity prediction.

To adapt the predictor to heterogeneous decomposed components, DBO is used to tune the key hyperparameters by minimizing validation MSE. The optimized hyperparameters and their corresponding search space are detailed in Table 3.

DBO uses a population size of 10 and runs for eight iterations. For predictor training, we set a maximum of 300 epochs and apply early stopping by monitoring validation loss (validation split =0.1, patience =30, restoring best weights).

#### 4.2.5. Implementation Details and Computational Environment

The proposed framework was implemented in TensorFlow 2.16.1 with Keras 3.5.0, and all models were optimized using the Adam optimizer. Prior to training, the input capacity sequences were normalized using Min–Max scaling. Computational resources. All experiments were executed on a unified computing platform (Intel(R) Core(TM) i5-12600KF CPU @ 3.70 GHz; 32 GB RAM).

### 4.3. Evaluation Metrics

To assess the prediction accuracy of the RUL prediction model, this study employs three evaluation metrics: the R^2^ score, Root Mean Squared Error (RMSE) and Mean Absolute Error (MAE) [34]. The mathematical formulations employed for computing these evaluation metrics are delineated below:

(26) $$RMSE=\sqrt{\frac{1}{n}{\sum_{i=1}^{n}\left(y_{i}-{\overset{\wedge }{y}}_{i}\right)}^{2}}$$

(27) $$MAE=\frac{1}{n}\sum_{i=1}^{n}\left|y_{i}-{\overset{\wedge }{y}}_{i}\right|$$

(28) $$R^{2}=1-\frac{\sum_{i=1}^{n}(y_{i}-{\overset{\wedge }{y}}_{i}{)}^{2}}{\sum_{i=1}^{n}(y_{i}-\bar{y}{)}^{2}}$$

where ${\hat{y}}_{i}$ is the predicted value of the *i*-th battery capacity; $y_{i}$ is the true value of the *i*-th battery capacity; $\bar{y}$ is the arithmetic mean of $y_{i}$.

In subsequent experiments, *R_RUL_* denotes the ground-truth measurement spanning from the prediction commencement point until the EOL cycle completion, whereas *P_RUL_* corresponds to the computationally estimated value over the identical operational interval. The precision assessment metric Absolute Error (AE), which quantifies the deviation magnitude between these two RUL parameters, is mathematically formulated as follows:

(29) $$AE=\left|P_{RUL}-R_{RUL}\right|$$

Pearson correlation coefficient quantifies the linear relationship between two datasets, ranging from −1 (perfect inverse correlation) to +1 (perfect positive correlation), with 0 indicating no linear association. This study computes r between decomposition residuals and original data to evaluate the trend preservation accuracy. Its formulation is

(30) $$r_{XY}=\frac{\sum_{i=1}^{n}\left(X_{i}-\bar{X})(Y_{i}-\bar{Y}\right)}{\sqrt{\sum_{i=1}^{n}{\left(X_{i}-\bar{X}\right)}^{2}}\sqrt{\sum_{i=1}^{n}{\left(Y_{i}-\bar{Y}\right)}^{2}}}$$

Concurrently, the orthogonality index (OI) measures the degree of independence between intrinsic mode functions (IMFs), calculated as the normalized cross-energy ratio of IMF pairs. Lower OI values indicate stronger mode separation and less information redundancy. It is defined as

(31) $${OI}_{jk}=\frac{\sum_{t=1}^{T}{IMF}_{j}(t)\times {IMF}_{k}(t)}{{\left\|{IMF}_{j}\right\|}_{2}\times {\left\|{IMF}_{k}\right\|}_{2}},\;\;j\neq k$$

where ${\left\|{IMF}_{j}\right\|}_{2}=\sqrt{\sum_{t=1}^{T}{\left[{IMF}_{j}(t)\right]}^{2}}$.

To quantitatively validate the realism of the HyT-GAN augmented sequences, we compute statistical consistency metrics between the real IMF samples and the generated samples for each IMF separately. Prior to calculation, both real and augmented sequences are normalized to $\left[-1,1\right]$ using Min–Max scaling. Let $x^{\mathrm{real}}=\{x_{t}^{\mathrm{real}}{\}}_{t=1}^{T}$ and $x^{\mathrm{aug}}=\{x_{t}^{\mathrm{aug}}{\}}_{t=1}^{T}$ denote the concatenated real and augmented sequences, respectively (after normalization). The mean and standard deviation are computed as

(32) $$\mu_{\mathrm{real}}=\frac{1}{T}\sum_{t=1}^{T}x_{t}^{\mathrm{real}}$$

(33) $$\mu_{\mathrm{aug}}=\frac{1}{T}\sum_{t=1}^{T}x_{t}^{\mathrm{aug}}$$

(34) $$\sigma_{\mathrm{real}}=\sqrt{\frac{1}{T}\sum_{t=1}^{T}(x_{t}^{\mathrm{real}}-\mu_{\mathrm{real}}{)}^{2}}$$

(35) $$\sigma_{\mathrm{aug}}=\sqrt{\frac{1}{T}\sum_{t=1}^{T}(x_{t}^{\mathrm{aug}}-\mu_{\mathrm{aug}}{)}^{2}}$$

The normalized mean shift is defined as

(36) $$\mathrm{Mean}\;\mathrm{shift}=\frac{|\Delta \mu |}{\sigma_{\mathrm{real}}}=\frac{|\mu_{\mathrm{aug}}-\mu_{\mathrm{real}}|}{\sigma_{\mathrm{real}}}$$

which measures the mean difference relative to the natural variability of the real IMF. The dispersion consistency is evaluated using the standard deviation ratio $\sigma_{\mathrm{aug}}/\sigma_{\mathrm{real}}$. To assess whether the temporal dependency structure is preserved, we compute the autocorrelation function (ACF) of $x^{\mathrm{real}}$ and $x^{\mathrm{aug}}$ up to a fixed maximum lag $L_{\mathrm{acf}}$ (set to 20 in this study). Denoting the ACFs as $\rho_{\mathrm{real}}(l)$ and $\rho_{\mathrm{aug}}(l)$ for $l=1,\ldots ,L_{\mathrm{acf}}$, the mean absolute ACF difference is defined as

(37) $$\mathrm{Mean}\;\mathrm{ACF}\;\mathrm{diff}=\frac{1}{L_{\mathrm{acf}}}\sum_{l=1}^{L_{\mathrm{acf}}}|\rho_{\mathrm{real}}(l)-\rho_{\mathrm{aug}}(l)|$$

where smaller values indicate that the augmented sequence better preserves the temporal correlation structure of the real IMF.

## 5. Results and Discussion

### 5.1. Signal Decomposition Comparison

**Figure 5:**
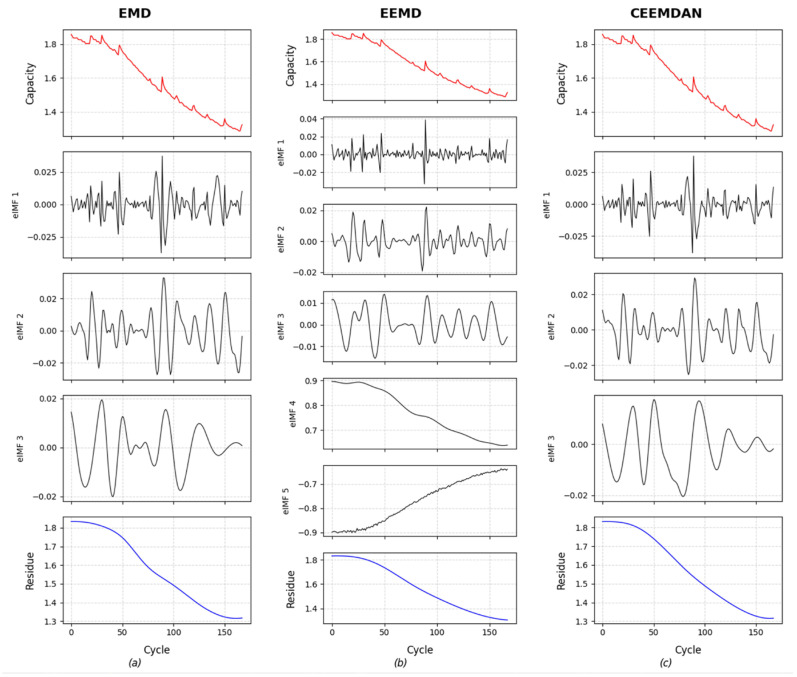
Exploded view of the capacity sequence of B0005: (**a**) EMD (**b**) EEMD (**c**) CEEMDAN.

To evaluate the relative merits of the selected signal processing techniques, EMD, EEMD, and CEEMDAN were applied for a comparative decomposition analysis of battery B0005. Figure 5 shows the multi-scale decomposition outcomes.

The primary limitation of conventional EMD manifests in Figure 5a as modal overlap issues, where IMF2 demonstrates spectral aliasing with adjacent IMF1 components. Furthermore, the residual term retains frequency elements characteristic of IMF3, indicating incomplete separation that undermines the physical significance of intrinsic mode functions and compromises decomposition fidelity. When transitioning to EEMD in Figure 5b, the white noise-assisted ensemble averaging effectively reduces mode mixing. However, this approach introduces new challenges of persistent noise contamination in higher-order IMFs due to incomplete cancelation during averaging processes. In contrast, the advanced CEEMDAN methodology presented in Figure 5c demonstrates dual advantages through its adaptive noise regulation framework, achieving the complete elimination of both modal interference and residual stochastic artifacts.

To further illustrate the effectiveness of CEEMDAN decomposition, this study computed the Pearson correlation coefficients between the residuals obtained from EMD, EEMD, and CEEMDAN decompositions and the original data, along with the OI between each IMF. Pearson correlation is used as an auxiliary indicator to evaluate trend preservation: by computing the correlation between the residual component and the original capacity series, we quantify whether the residual retains the dominant long-term degradation tendency after decomposition. A higher Pearson value suggests that the decomposition produces a residual that is more consistent with the global trend of the original signal, while separating short-term fluctuations (including noise and regeneration-related variations) into IMFs. In contrast, the OI is adopted to evaluate the mode separability among IMFs: lower OI values indicate weaker inter-mode coupling and less information redundancy, implying that the decomposed IMFs are more independent and thus more suitable for subsequent component-wise modeling and reconstruction.

Validation was performed using the NASA B0005 dataset and the CALCE CS235 dataset, with the results presented in Table 4.

As can be observed, on the B0005 dataset, the differences in Pearson correlation coefficients between the decomposition residuals and the original data are minimal; all three methods are capable of accurately capturing the trend of battery capacity degradation. EEMD yielded the lowest OI among its IMFs, followed by CEEMDAN. On the CALCE CS235 dataset, CEEMDAN outperformed the other two methods in both the Pearson correlation coefficient and OI. These results demonstrate the effectiveness and robustness of the CEEMDAN decomposition.

### 5.2. Augmented Data Validation

To verify that the HyT-GAN-generated samples are statistically consistent with the real CEEMDAN-decomposed components, we conducted a quantitative validation on the NASA B0005 dataset by comparing the first- and second-order statistics and the temporal dependency structure between the real and augmented sequences for each IMF. Specifically, we report the mean $\left(\mu \right)$ and standard deviation $\left(\sigma \right)$ of real versus augmented data, the normalized mean shift $|\Delta \mu |/\sigma_{\mathrm{real}}$, the standard deviation ratio $\sigma_{\mathrm{aug}}/\sigma_{\mathrm{real}}$, and the mean absolute ACF difference over a fixed lag window.

As shown in Table 5, the augmented data exhibit small mean shifts across all IMFs, with $|\Delta \mu |/\sigma_{\mathrm{real}}$ ranging from 0.031 to 0.114, indicating that the generator does not introduce substantial bias relative to the natural variability of the real components. Meanwhile, the dispersion level is well preserved, with $\sigma_{\mathrm{aug}}/\sigma_{\mathrm{real}}$ close to 1 (from 0.990 to 1.154), suggesting that HyT-GAN maintains comparable fluctuation intensity and avoids mode collapse. In addition, the temporal correlation structure is largely retained: the mean ACF diff remains low (from 0.043 to 0.231), especially for the low-frequency component (IMF4), implying that the generated sequences preserve the key autocorrelation patterns of the real degradation-related signals. Overall, these statistical results support that HyT-GAN produces realistic augmented samples that are consistent with the original IMF distributions and temporal dependencies, providing reliable additional training data for early-stage RUL prediction.

### 5.3. Hyperparameter Optimization

The hybrid method proposed in this article indicates that CEEMDAN-decomposed components exhibit heterogeneous temporal characteristics. The high-frequency IMFs (e.g., IMF1–2) are dominated by rapid fluctuations and noise-sensitive local variations, whereas lower-frequency components (e.g., IMF3–4) mainly reflect smoother long-term degradation dynamics with stronger temporal dependence. Consequently, different IMF components require different model capacities and training configurations in the CNN-BiGRU predictor; a single static hyperparameter setting is suboptimal and may lead to unstable performance in early-stage RUL prediction. Therefore, we employ the DBO algorithm to optimize the key hyperparameters of the CNN-BiGRU model for each IMF separately, including the Conv1D filter number, BiGRU hidden units, batch size, and dropout rate. The optimized results in Table 6 show clear IMF-wise variability in these hyperparameters, confirming that adaptive hyperparameter selection is necessary to accommodate the diverse frequency contents and noise levels across decomposed components and to improve early-stage prediction robustness.

**Figure 6:**
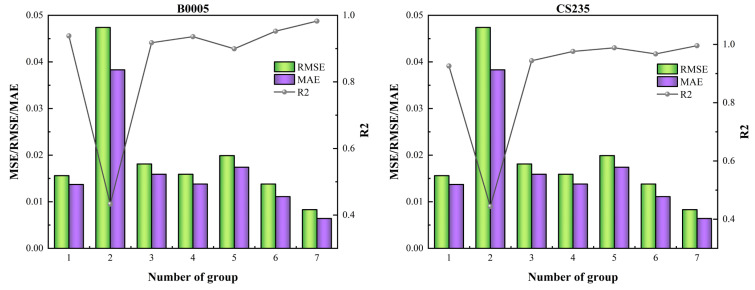
Predictors with different hyperparameter combinations.

In order to verify the effectiveness of the DBO algorithm, 50% of the historical data was used for training. The CNN-BiGRU model applied to different datasets was optimized and compared with models using static hyperparameter configurations. Table 7 presents the chosen combinations of static hyperparameter configurations which include the Baseline group, Extreme Config group, CNN Filters Focus group, Random Search group, Overfitting-Oriented group, and Self-Adjusted group. The prediction errors obtained are shown in Figure 6. The seventh group is the DBO algorithm tuning group. The DBO algorithm is used to optimize the parameters. The iteration number is set to 8, and the population number is 10. It can be seen from Figure 6 that the parameter combination of the DBO-optimized CNN-BiGRU model has a higher prediction accuracy than the fixed hyperparameter combination.

### 5.4. Comparative Analysis of RUL Prediction Results

In practical battery management systems, RUL estimation is driven by sensor-acquired time series signals (e.g., voltage, current, temperature, and capacity). These measurements are typically affected by noise, environmental disturbances, sensor drift, and operational variability, leading to degradation trajectories that are highly nonlinear and non-stationary, especially in early-life stages. Under such conditions, shallow machine learning models often rely on manual feature engineering and implicit stationarity assumptions, which limits their ability to capture multi-scale temporal dependencies and long-range degradation patterns.

In contrast, deep models can learn hierarchical temporal representations directly from raw sequences, enabling more effective extraction of degradation signatures in the presence of noise and non-stationarity. Moreover, our framework is specifically designed to address early-stage data scarcity and regeneration-induced fluctuations by combining CEEMDAN-based multi-scale decomposition and HyT-GAN augmentation before forecasting. This design improves robustness and generalization in small-sample settings, where shallow models are typically more sensitive to data insufficiency and distribution shifts. Finally, while deep models can be more computationally demanding during training, training can be performed offline, and online inference can be executed efficiently; thus, the accuracy–cost trade-off is favorable for sensor-driven battery health monitoring applications.

**Figure 7:**
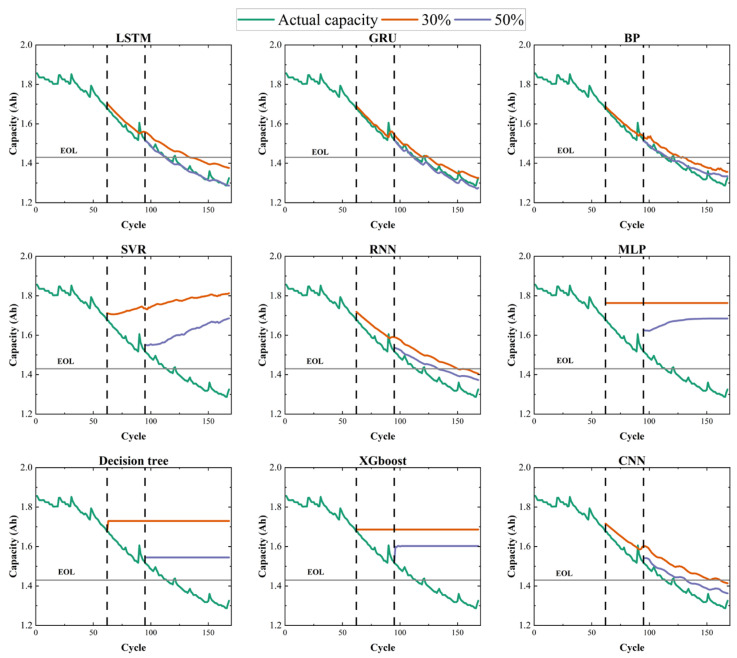
Prediction results of mainstream regression algorithm based on B0005 dataset. The dashed line indicates the end-of-life (EOL) failure threshold.

In this paper, representative shallow regression models and commonly used sequence models for battery RUL prediction were evaluated on the NASA dataset (B0005). As shown in Figure 7, under the early-stage setting with only 30% historical data, most baseline methods exhibit unstable forecasts and often fail to capture the correct degradation trend, highlighting the strong nonlinearity and non-stationarity of sensor-acquired degradation trajectories and the difficulty of small-sample learning. Even when the training portion increases to 50%, several methods still struggle to produce a reliable capacity degradation trend, indicating that early RUL prediction remains challenging for mainstream approaches.

Table 8 summarizes the quantitative results. Among the baselines, GRU and LSTM achieve the best performance under 30% and 50% training data, respectively. However, the proposed hybrid framework consistently outperforms these models, and its accuracy with only 30% historical data already exceeds that of the best baseline trained with 50% data. This superiority supports our motivation for using a higher-capacity deep framework with decomposition and augmentation modules to improve robustness and generalization in early-stage, small-sample scenarios.

**Figure 8:**
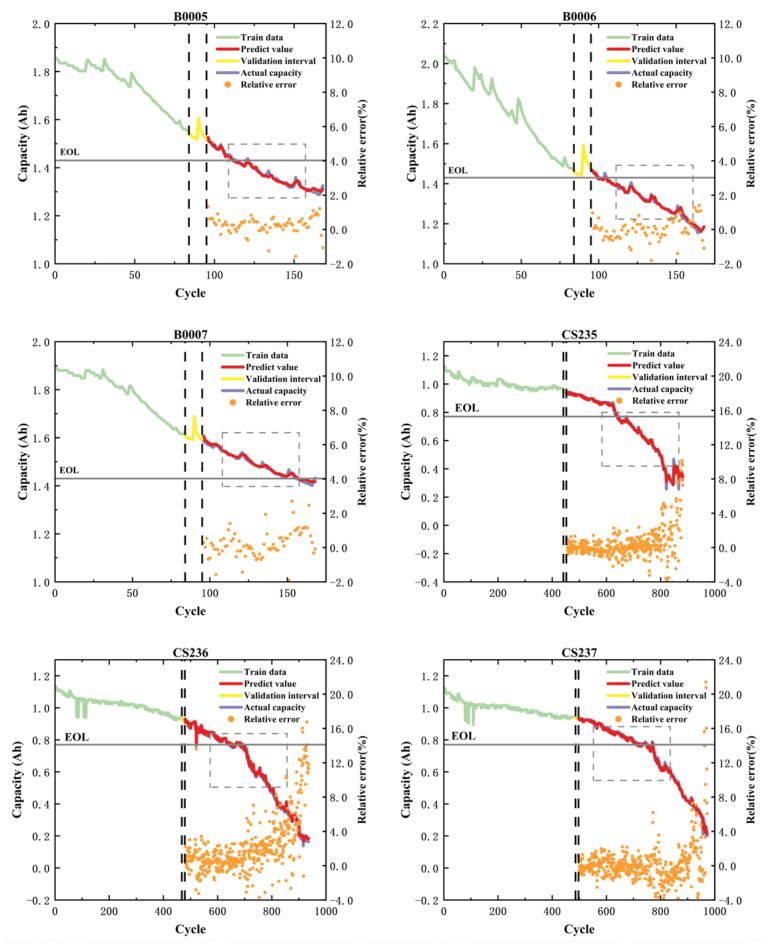
Degradation forecasting results with 50% historical data (NASA and CALCE). The gray line indicates the end-of-life (EOL) failure threshold.

Regarding the currently widely used 50% of historical data, the RUL prediction using the proposed hybrid model architecture is illustrated in Figure 8. In the figure, the dashed boxes indicate the capacity regeneration phenomenon. Both the NASA and CALCE datasets reveal that the prognostic trajectories maintain precise synchronization with the authentic degradation trends, while effectively capturing capacity rebound characteristics induced by electrochemical noise and cyclic regeneration phenomena. This indicates that the proposed hybrid model can achieve accurate predictive results across different datasets, demonstrating the CEEMDAN decomposition method’s effectiveness for capacity regeneration. Table 9 presents the predictive results of the hybrid model across various datasets. As evidenced in the table, the proposed method achieves precise evaluation metrics across diverse datasets, with all RUL prediction errors confined within two cycles. It shows that the model has high prediction accuracy and strong cross-dataset generalizability on different datasets.

**Figure 9:**
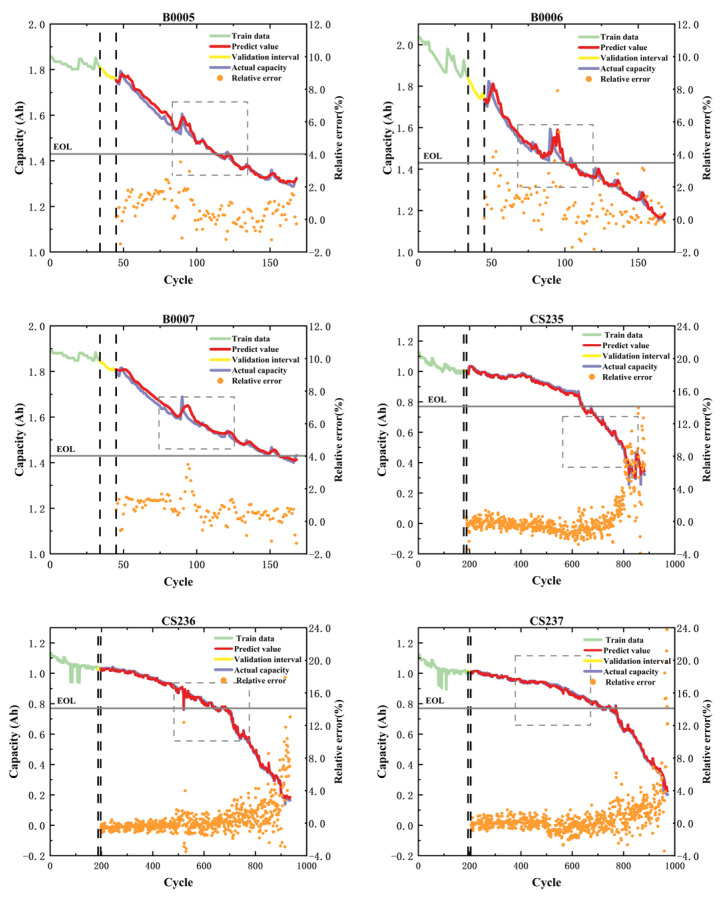
Degradation forecasting results with 20% historical data (NASA and CALCE). The gray line indicates the end-of-life (EOL) failure threshold.

To validate the early-stage prognostic capability under data scarcity constraints, the historical data used for training were reduced to 20% of the overall capacity data. As shown in Figure 9, the results indicate that this method can accurately capture the trend of capacity decline using only 20% of the historical capacity data. It can also accurately reflect this trend in the presence of significant capacity regeneration phenomena, a feature not possessed by other methods that utilize a single historical capacity data input in the current research. As shown in Table 10, the average RMSE values are 0.0212 (NASA) and 0.0136 (CALCE), with R^2^ metrics exceeding 0.985 across all test cases except B0006. Remarkably, these metrics rival the performance of comparative methods requiring 50–70% training data inputs. Furthermore, Absolute Error distributions across all experimental configurations remain bounded within 3% tolerance thresholds, empirically confirming the framework’s competence in data-constrained prognostic scenarios.

**Figure 10:**
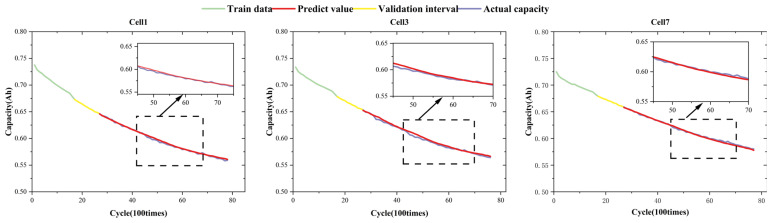
Early-stage RUL prediction results on the Oxford Dataset using 20% historical capacity data.

To further assess cross-scenario generalization beyond the NASA and CALCE benchmarks, we additionally evaluated the proposed framework on the Oxford Battery Degradation Dataset 1. In this study, Cells 1, 3, and 7 were selected, and we adopted the same early-stage protocol by using only the first 20% of the historical capacity trajectory as the prediction starting point. The result is shown in Figure 10. Despite the differences in cell form factor, nominal capacity, temperature, and dynamic load profile compared with the constant-current cycling conditions in NASA/CALCE, the proposed CEEMDAN–HyT-GAN–CNN-BiGRU framework continues to produce consistent degradationbtrend tracking on these Oxford cells, indicating that the method is not restricted to a single dataset or testing protocol and exhibits promising cross-scenario applicability.

**Figure 11:**
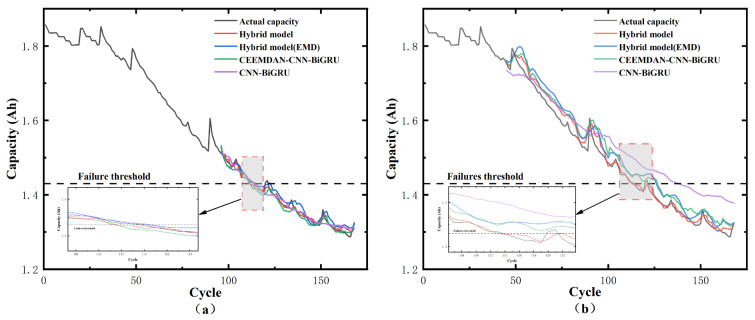
Prediction results of ablation study: (**a**) 50% historical data; (**b**) 20% historical data.

To assess the efficacy of each strategy in the proposed framework, four ablation configurations were evaluated on the NASA B0005 battery: (1) CNN-BiGRU, (2) CEEMDAN–CNN-BiGRU, (3) EMD–HyT-GAN–DBO–CNN-BiGRU, and (4) CEEMDAN–HyT-GAN–DBO–CNN-BiGRU. As reported in Table 11, the standalone CNN-BiGRU achieves acceptable performance when trained with 50% historical capacity data; however, when the available history is reduced to 20%, its prediction accuracy degrades sharply and the AE exhibits large fluctuations (e.g., AE = 24). This behavior indicates that the baseline CNN-BiGRU is highly sensitive to data scarcity, leading to unstable forecasts and larger prediction variance under limited samples. After introducing CEEMDAN, the prediction becomes more stable because decomposition separates multi-scale trend and fluctuation components, enabling the model to better capture capacity regeneration patterns. When CEEMDAN is replaced by EMD, the performance decreases, since EMD is less effective at separating high-frequency noise from low-frequency degradation trends, which degrades the quality of the decomposed components. Finally, incorporating HyT-GAN augmentation further improves robustness in the 20% setting by increasing the sample diversity and stabilizing training, resulting in consistently lower errors and demonstrating the necessity of the proposed components for reliable early-stage RUL prediction. The ablation experiment results are shown in Figure 11.

**Figure 12:**
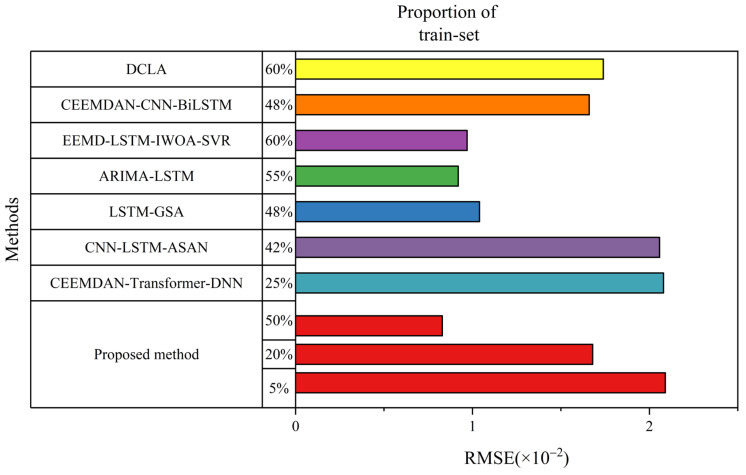
Comparison of RUL prediction results for batteries B0005.

The above experiments demonstrate that the early life prediction method proposed in this paper achieves high accuracy even when using a significantly smaller amount of data (20%) compared to traditional methods. To further investigate the performance of the hybrid model under minimal samples, we conducted predictions using only 8% of the historical data on the NASA lithium-ion battery B0005 dataset. The results indicate that the model achieves an accuracy of RMSE = 0.0209 and MAE = 0.0158 using merely 8% of the data. As illustrated in Figure 12, the RUL prediction results of the proposed hybrid method are compared with several references from current studies utilizing the NASA dataset. These studies employ various novel hybrid methods for battery RUL prediction, including the CEEMDAN–Transformer–DNN [18], CEEMDAN–CNN–BiLSTM [44], EEMD–LSTM–IWOA–SVR [45], ARIMA–LSTM [46], LSTM–GSA [47], CNN–LSTM–ASAN [48], and DCLA [49]. The proportion of training data used in these studies ranges from 48% to 60%. Compared to the prediction methods shown in Figure 12, the proposed hybrid method yields the smallest RMSEs. Under small-sample training conditions, it incurs an acceptable range of accuracy loss relative to the current literature on the NASA Ames PCoE battery dataset, while significantly reducing the amount of training data required. This highlights the model’s excellent data efficiency and its capability to extract critical aging information from very early cycles.

## 6. Conclusions

This paper proposes a hybrid machine learning model with time series augmentation to predict the RUL of LIBs, aimed at addressing the accuracy issues in early RUL prediction caused by noise interference, capacity regeneration phenomena, and insufficient data in traditional methods. The CEEMDAN algorithm decomposes the original capacity sequence, effectively separating high-frequency oscillatory components from low-frequency trend components, thereby significantly suppressing capacity regeneration issues. Combined with the HyT-GAN model based on the Transformer module, the self-attention mechanism performs high-fidelity time series data augmentation on the decomposed components, overcoming the limitations of sparse historical data in early-stage predictions. The CNN-BiGRU model effectively captures the complex patterns in battery degradation through local feature extraction and global dependency modeling. The application of the Dung Beetle Optimization algorithm achieves adaptive hyperparameter tuning in the model, leading to substantial enhancements in both predictive accuracy and generalization performance.

In order to evaluate the generalizability of the proposed method, validation was conducted by employing publicly available datasets obtained from the NASA and CALCE repositories. The prediction error (RMSE < 0.016, R^2^ > 0.976) of this method is significantly better than that of mainstream models such as LSTM and GRU under 50% historical data. In the early prediction scenario using only 20% of the historical data, the model still maintains high accuracy (RMSE ≤ 0.0296, R^2^ ≥ 0.9643) and verifies its robustness under the condition of data scarcity. Finally, ablation experiments verified the effectiveness of the hybrid model’s strategies.

Although the model performs excellently on the existing dataset, its generalizability capability under complex conditions still requires further validation. Moreover, the processing requirements inherent in the current modeling approach present notable challenges. The development of combined forecasting methodologies leveraging multimodal sensor data—voltage, current, and temperature—emerges as a pivotal research domain for optimizing the prognostic accuracy in RUL assessment systems.

## Figures and Tables

**Table 1:** Details of NASA datasets.

Battery	Discharge Current	Rated Capacity	Charging/Discharge Cut-Off Voltage	Minimal Charge Current	Failure Threshold
B0005	2 A	2 Ah	4.2/2.7 V	20 mA	1.43 Ah
B0006	2 A	2 Ah	4.2/2.7 V	20 mA	1.43 Ah
B0007	2 A	2 Ah	4.2/2.7 V	20 mA	1.43 Ah

**Table 2:** Details of CALCE datasets.

Battery	Discharge Current	Rated Capacity	Charging/Discharge Cut-Off Voltage	Minimal Charge Current	Failure Threshold
CS2_35	1.1 A	1.1 Ah	4.2/2.7 V	50 mA	0.77 Ah
CS2_36	1.1 A	1.1 Ah	4.2/2.7 V	50 mA	0.77 Ah
CS2_37	1.1 A	1.1 Ah	4.2/2.7 V	50 mA	0.77 Ah

**Table 3:** Hyperparameter search space.

Hyperparameters	Lower Bound	Upper Bound
Dropout rate	0.01	0.6
Batch size	1	60
CNN filters	10	600
GRU units	10	600

**Table 4:** Residual-data correlation and IMF orthogonality for battery datasets using different decomposition methods.

Battery	Method	Pearson Coefficients	Orthogonality Index
B0005	CEEMDAN	0.9972	0.1299
	EEMD	0.9971	0.1108
	EMD	0.9970	0.1969
CS235	CEEMDAN	0.9714	0.0801
	EEMD	0.9332	0.0983
	EMD	0.9189	0.0948

**Table 5:** Statistical Consistency Analysis Between Real and HyT-GAN-Generated IMF Components (NASA B0005).

IMF	μ_real_	μ_aug_	σ_real_	σ_aug_	Mean Shift	Std Ratio	Mean ACF Diff
1	−0.015106	0.012345	0.241474	0.272951	0.114	1.130	0.173
2	−0.091342	−0.072584	0.359597	0.356120	0.052	0.990	0.231
3	−0.011633	−0.019050	0.241588	0.278687	0.031	1.154	0.161
4	0.137840	0.205693	0.651033	0.677545	0.104	1.041	0.043

**Table 6:** DBO-optimized CNN-BiGRU hyperparameters for individual IMF components (NASA B0005).

IMF	Dropout Rate	Batch Size	CNN Filters	GRU Units
1	0.2885	60	12	600
2	0.6	45	600	600
3	0.0306	17	416	459
4	0.6	13	562	600

**Table 7:** Fixed hyperparameter combination settings.

	Group	Dropout Rate	Batch Size	CNN Filters	GRU Units
1	Baseline	0.2	32	64	128
2	Extreme Config	0.6	1	8	16
3	CNN Filters Focus	0.2	32	256	128
4	Random Search	0.35	47	183	294
5	Overfitting-Oriented	0.1	60	512	512
6	Self-Adjusted	0.3	25	256	256

**Table 8:** The performance metrics of conventional regression modeling approaches in forecasting tasks on dataset B0005 under partial historical data inputs of 30% and 50% proportions.

Method	LSTM [35]		GRU [36]		RNN [37]		SVR [38]		CNN [39]	
	30%	50%	30%	50%	30%	50%	30%	50%	30%	50%
R2	0.7398	0.9568	0.9392	0.9321	0.4896	0.4412	−7.908	−19.27	0.4292	0.5323
RMSE	0.0573	0.0136	0.0277	0.0171	0.0853	0.0576	0.3348	0.2958	0.0619	0.0377
MAE	0.0529	0.0082	0.0253	0.0129	0.0809	0.0529	0.3155	0.2831	0.0583	0.0347
Method	BP [40]		MLP [41]		Decision tree [42]		XGboost [43]		Proposed method	
	30%	50%	30%	50%	30%	50%	30%	50%	30%	50%
R2	0.9294	0.8869	−8.523	−14.05	−5.181	−3.642	−4.513	−11.01	0.9808	0.9825
RMSE	0.0394	0.0259	0.3463	0.2549	0.2969	0.1661	0.2635	0.2276	0.0153	0.0083
MAE	0.0338	0.0201	0.3145	0.2301	0.2723	0.1493	0.2384	0.2170	0.0117	0.0064

**Table 9:** Prediction accuracy of proposed method for different datasets under 50% historical data.

Battery	R^2^	RMSE	MAE	RUL	PRUL	AE
B0005	0.9825	0.0083	0.0064	18	17	1
B0006	0.9832	0.0108	0.0080	5	4	1
B0007	0.9768	0.0079	0.0064	60	61	1
CS235	0.9958	0.0130	0.0070	187	187	0
CS236	0.9952	0.0160	0.0118	165	167	2
CS237	0.9965	0.0119	0.0083	214	212	2

**Table 10:** Prediction accuracy of proposed method for different datasets under 20% historical data.

Battery	R^2^	RMSE	MAE	RUL	PRUL	AE
B0005	0.9867	0.0168	0.0129	69	67	2
B0006	0.9643	0.0296	0.0198	56	56	0
B0007	0.9858	0.0172	0.0133	110	111	1
CS235	0.9958	0.0135	0.0089	452	448	4
CS236	0.9972	0.0137	0.0107	448	441	7
CS237	0.9957	0.0136	0.0095	506	513	7

**Table 11:** Prediction results of ablation study on B0005 dataset.

Method	Degradation Data	R^2^	RMSE	MAE	AE
CNN-BiGRU	50%	0.9607	0.0125	0.0092	1
	20%	0.8537	0.0567	0.0502	24
CEEMDAN–CNN-BiGRU	50%	0.9673	0.0114	0.0089	3
	20%	0.9536	0.0307	0.0283	16
EMD–HyT-GAN–DBO–CNN-BiGRU	50%	0.9587	0.0128	0.0111	1
	20%	0.9477	0.0326	0.0298	15
CEEMDAN–HyT-GAN–DBO–CNN-BiGRU	50%	0.9825	0.0083	0.0064	2
	20%	0.9867	0.0168	0.0129	2

## Data Availability

The data used in this paper are openly available. NASA dataset: https://www.nasa.gov/content/prognostics-center-of-excellence-data-set-repository (accessed on 8 May 2025); CALCE dataset: https://calce.umd.edu/data#CS2 (accessed on 8 May 2025); Oxford Battery Dataset: https://ora.ox.ac.uk/objects/uuid:03ba4b01-cfed-46d3-9b1a-7d4a7bdf6fac (accessed on 6 January 2026).

## References

[1] Contestabile M., Offer G., Slade R., Jaeger F., Thoennes M. (2011). Battery electric vehicles, hydrogen fuel cells and biofuels. Which will be the winner?. Energy Environ. Sci..

[2] Thackeray M.M., Wolverton C., Isaacs E.D. (2012). Electrical energy storage for transportation—Approaching the limits of, and going beyond, lithium-ion batteries. Energy Environ. Sci..

[3] Tang X.P., Liu K.L., Wang X., Gao F.R., Macro J., Widanage W.D. (2020). Model Migration Neural Network for Predicting Battery Aging Trajectories. IEEE Trans. Transp. Electrif..

[4] Laadjal K., Cardoso A.J.M. (2021). Estimation of Lithium-Ion Batteries State-Condition in Electric Vehicle Applications: Issues and State of the Art. Electronics.

[5] Li S., Fang H.J., Shi B. (2021). Remaining useful life estimation of Lithium-ion battery based on interacting multiple model particle filter and support vector regression. Reliab. Eng. Syst. Saf..

[6] Shu X., Shen S.Q., Shen J.W., Zhang Y.J., Li G., Chen Z., Liu Y.G. (2021). State of health prediction of lithium-ion batteries based on machine learning: Advances and perspectives. Iscience.

[7] Mao J.L., Miao J.Z., Lu Y.Y., Tong Z.M. (2021). Machine learning of materials design and state prediction for lithium ion batteries. Chin. J. Chem. Eng..

[8] Miguel E., Plett G.L., Trimboli M.S., Oca L., Iraola U., Bekaert E. (2021). Review of computational parameter estimation methods for electrochemical models. J. Energy Storage.

[9] Chen L., An J.J., Wang H.M., Zhang M., Pan H.H. (2020). Remaining useful life prediction for lithium-ion battery by combining an improved particle filter with sliding-window gray model. Energy Rep..

[10] Vichard L., Ravey A., Venet P., Harel F., Pelissier S., Hissel D. (2021). A method to estimate battery SOH indicators based on vehicle operating data only. Energy.

[11] Chen L.P., Xie S.Q., Lopes A.M., Li H.F., Bao X.Y., Zhang C.L., Li P.H. (2024). A new SOH estimation method for Lithium-ion batteries based on model-data-fusion. Energy.

[12] Zhang Y.W., Tang Q.C., Zhang Y., Wang J.B., Stimming U., Lee A.A. (2020). Identifying degradation patterns of lithium ion batteries from impedance spectroscopy using machine learning. Nat. Commun..

[13] Zhang X.W., Qin Y., Yuen C., Jayasinghe L., Liu X. (2021). Time-Series Regeneration With Convolutional Recurrent Generative Adversarial Network for Remaining Useful Life Estimation. IEEE Trans. Ind. Inform..

[14] Shi C., Zhu D., Zhang L., Song S., Sheldon B.W. (2024). Transfer learning prediction on lithium-ion battery heat release under thermal runaway condition. Nano Res. Energy.

[15] Hu W.Y., Zhao S.S. (2022). Remaining useful life prediction of lithium-ion batteries based on wavelet denoising and transformer neural network. Front. Energy Res..

[16] Cheng G., Wang X.Z., He Y.R. (2021). Remaining useful life and state of health prediction for lithium batteries based on empirical mode decomposition and a long and short memory neural network. Energy.

[17] Wang G., Sun L.F., Wang A.J., Jiao J.F., Xie J.L. (2024). Lithium battery remaining useful life prediction using VMD fusion with attention mechanism and TCN. J. Energy Storage.

[18] Cai Y.X., Li W.M., Zahid T., Zheng C.H., Zhang Q.G., Xu K. (2023). Early prediction of remaining useful life for lithium-ion batteries based on CEEMDAN-transformer-DNN hybrid model. Heliyon.

[19] Ma G.J., Wang Z.D., Liu W.B., Fang J.Z., Zhang Y., Ding H., Yuan Y. (2023). A two-stage integrated method for early prediction of remaining useful life of lithium-ion batteries?. Knowl.-Based Syst..

[20] Tong Z.M., Miao J.Z., Tong S.G., Lu Y.Y. (2021). Early prediction of remaining useful life for Lithium-ion batteries based on a hybrid machine learning method. J. Clean. Prod..

[21] Severson K.A., Attia P.M., Jin N., Perkins N., Jiang B., Yang Z., Chen M.H., Aykol M., Herring P.K., Fraggedakis D. (2019). Data-driven prediction of battery cycle life before capacity degradation. Nat. Energy.

[22] Zhang Y.Z., Xiong R., He H.W., Pecht M.G. (2018). Long Short-Term Memory Recurrent Neural Network for Remaining Useful Life Prediction of Lithium-Ion Batteries. IEEE Trans. Veh. Technol..

[23] Liang Y.Q., Zhao S. (2024). Early Prediction of Remaining Useful Life for Lithium-Ion Batteries with the State Space Model. Energies.

[24] Lv K., Ma Z.Q., Bao C., Liu G.C. (2024). Indirect Prediction of Lithium-Ion Battery RUL Based on CEEMDAN and CNN-BiGRU. Energies.

[25] Zhang C.L., He Y.G., Yuan L.F., Xiang S. (2017). Capacity Prognostics of Lithium-Ion Batteries using EMD Denoising and Multiple Kernel RVM. IEEE Access.

[26] Mao L., Xu J., Chen J.J., Zhao J.B., Wu Y.B., Yao F.J. (2020). A LSTM-STW and GS-LM Fusion Method for Lithium-Ion Battery RUL Prediction Based on EEMD. Energies.

[27] Goodfellow I.J., Pouget-Abadie J., Mirza M., Xu B., Warde-Farley D., Ozair S., Courville A., Bengio Y. (2014). Generative adversarial nets. Adv. Neural Inf. Process. Syst..

[28] Xue J.K., Shen B. (2023). Dung beetle optimizer: A new meta-heuristic algorithm for global optimization. J. Supercomput..

[29] Wu Y.Y., Xu Y., Huang X.D. (2025). Wind Power Prediction Model based on Integrated Osprey and Adaptive T-distribution Dung Beetle Optimization Algorithm. J. Bionic Eng..

[30] Saha B., Goebel K. (2007). Battery data set. NASA AMES Prognostics Data Repository.

[31] Vasan A.S.S., Mahadeo D.M., Doraiswami R., Huang Y., Pecht M. (2013). Point-of-care biosensor system. Front. Biosci..

[32] Birkl C. (2017). Diagnosis and Prognosis of Degradation in Lithium-Ion Batteries. Doctoral Dissertation.

[33] Hu X.S., Xu L., Lin X.K., Pecht M. (2020). Battery Lifetime Prognostics. Joule.

[34] Zhao L.L., Song S.T., Wang P.Y., Wang C.Y., Wang J.J., Guo M.Z. (2024). A MLP-Mixer and mixture of expert model for remaining useful life prediction of lithium-ion batteries. Front. Comput. Sci..

[35] Hochreiter S., Schmidhuber J. (1997). Long short-term memory. Neural Comput..

[36] Cho K., Van Merriënboer B., Bahdanau D., Bengio Y. (2014). On the properties of neural machine translation: Encoder-decoder approaches. arXiv.

[37] Zaremba W., Sutskever I., Vinyals O. (2014). Recurrent neural network regularization. arXiv.

[38] Smola A.J., Schölkopf B. (2004). A tutorial on support vector regression. Stat. Comput..

[39] O’Shea K., Nash R. (2015). An introduction to convolutional neural networks. arXiv.

[40] Hecht-Nielsen R. (1992). Theory of the backpropagation neural network. Neural Networks for Perception.

[41] Murtagh F. (1991). Multilayer perceptrons for classification and regression. Neurocomputing.

[42] Song Y.-Y., Lu Y. (2015). Decision tree methods: Applications for classification and prediction. Shanghai Arch. Psychiatry.

[43] Chen T., Guestrin C. Xgboost: A scalable tree boosting system. Proceedings of the 22nd ACM SIGKDD International Conference on Knowledge Discovery and Data Mining.

[44] Guo X.F., Wang K.Z., Yao S., Fu G.J., Ning Y. (2023). RUL prediction of lithium ion battery based on CEEMDAN-CNN BiLSTM model. Energy Rep..

[45] Gao K.P., Sun J.J., Huang Z.Y., Liu C.Q. (2024). Capacity prediction of lithium-ion batteries based on ensemble empirical mode decomposition and hybrid machine learning. Ionics.

[46] Wang Y.Z., Hei C.Y., Liu H., Zhang S.D., Wang J.G. (2023). Prognostics of Remaining Useful Life for Lithium-Ion Batteries Based on Hybrid Approach of Linear Pattern Extraction and Nonlinear Relationship Mining. IEEE Trans. Power Electron..

[47] Reza M.S., Hannan M.A., Mansor M.B., Ker P.J., Tiong S.K., Hossain M.J. (2024). Gravitational Search Algorithm Based LSTM Deep Neural Network for Battery Capacity and Remaining Useful Life Prediction With Uncertainty. IEEE Trans. Ind. Appl..

[48] Li Y.M., Qin X.J., Ma F.R., Wu H.R., Chai M., Zhang F.J., Jiang F.H., Lei X. (2024). Fusion Technology-Based CNN-LSTM-ASAN for RUL Estimation of Lithium-Ion Batteries. Sustainability.

[49] Xia T.C., Zhang X., Zhu H.F., Zhang X.C., Shen J. (2023). An accurate denoising lithium-ion battery remaining useful life prediction model based on CNN and LSTM with self-attention. Ionics.

